# Bone Marrow-Derived Mesenchymal Stem Cells: Current and Future Applications in the Urinary Bladder

**DOI:** 10.4061/2010/765167

**Published:** 2011-01-03

**Authors:** Beth A. Drzewiecki, John C. Thomas, Stacy T. Tanaka

**Affiliations:** Division of Pediatric Urology, Monroe Carell, Jr. Children's Hospital, Vanderbilt University Medical Center, Nashville, TN 37232, USA

## Abstract

Mesenchymal stem cells can be isolated from almost any adult tissue. In this paper we focus on bone marrow-derived mesenchymal stem cells which have captured the interest of researchers since their introduction because of the promising potential of tissue regeneration and repair. They are known for their ability to self-renew and differentiate into diverse lineages while maintaining low immunogenicity. The exact mechanisms behind how these cells work still remain unclear, and there is a continuing shift in the paradigms that support them. There has been extensive research in multiple organ systems; however, the genitorurinary system has been vastly underrepresented. This article discusses the background behind bone marrow-derived mesenchymal stem cells and they are currently being applied to the urinary bladder in the realm of tissue engineering. We also postulate on their future applications based on the current literature in other organ systems.

## 1. Introduction

Bone marrow mesenchymal stem cells (MSCs) were first identified in the 1960s by Ernest A McCulloch and James E. Till as being a clonal source of cells for further use [1]. Further experiments in the 1970s and 80s by Friedenstein et al. expanded upon the potential of MSCs by demonstrating their capacity for self-renewal and multilineage differentiation [2, 3]. In the ensuing decades extensive research has gone into unlocking the therapeutic potential for MSCs.

Stem cells are defined by their potency, the capacity to differentiate into a variety of cells, and cell lineages. Embryonic stem cells, as their name implies, are cultured from the inner cell mass of blastocysts during early embryonic development and have received a good deal of attention due to their potential for misuse and ethical consideration [4]. These cells are undifferentiated at the time of harvest and therefore are pluripotent with the ability to develop into any cell type. MSCs are an adult stem cell isolated not only from bone marrow but also from most adult tissue including adipose, liver, amniotic fluid, lung, skeletal muscle, and kidney. However, one advantage of bone marrow is the ease with which these cells are cultured. Furthermore, their differentiation into osteocytes, adipocytes, chondrocytes, hepatocytes, and myocytes has been extensively characterized, with the further possibility of differentiation into cardiomyocytes and neurons [5–8]. Even more recent investigations suggest that MSCs can differentiate into endodermal lineage as well [7, 9].

There are many reasons why researchers have been enamored by MSCs. Even though only a small percentage of cells in the bone marrow are MSCs, they are easily isolated because of their affinity and adherence to plastic [3, 8]. Even a small number of MSC can multiply into millions of cells under the right culture conditions. Extensive protocols have been delineated for culturing stem cells in a variety of animals including humans. MSCs do not express MHC II rendering them nonimmunogenic, thus precluding the need for lifelong immunosuppression with allogenic transplantation [7]. Also, intravenous injection allows for safe, fast, and easy transplantation of,F MSCs into the host [10]. 

There are currently 119 clinical trials that have or will involve MSCs (http://www.clinicaltrials.gov/). While there have been great strides in the translation of the bench research into clinical practice, there is still very little understood about the exact nature of the MSCs that are being described. There are currently no universal markers for identifying MSCs or characterizing their subpopulations and thus a standardization process has been difficult [11, 12]. Furthermore, there seems to be a discrepancy between the behavior of* in vitro* expanded MSCs and fresh, nonmanipulated MSCs and their microenvironmental interactions [13].

### 1.1. Proposed Mechanisms of MSCs for Repair and Regeneration

Because MSCs were noted to differentiate into various cell lines, it was thought initially that the mechanism in which MSCs acted was through engraftment and differentiation into the injured tissue. In fact engrafted MSCs have been identified at sites of injury in lung [14], liver [15], heart [10], kidney [16], and brain [17] with an associated improvement in function. There have been numerous reports of systemic infusions of MSCs leading to functional improvements based on this paradigm of engraftment and differentiation; however, it has been challenged as clinically relevant [14, 18]. It is not only difficult to demonstrate extensive engraftment of cells, but also many cells are trapped in the lungs after systemic injection [10, 18]. Furthermore, prolonged responses of therapeutic effect were noted after identification of MSCs had ceased. Thus, it became evident that another mechanism in which MSCs exerted their reparative benefit existed.

Extensive research has been performed showing MSCs ability to remold damaged cardiac tissue after myocardial infarction [10], improve fibrotic responses in lung disease [19], and facilitate repair of spinal cord [20], bony and cartilaginous injury. The variety in the underlying nature of the aforementioned injuries (ischemia, fibrosis, fracture, etc.) exemplifies that the fundamental basis of MSCs in a repair model is their ability to identify the site of injury via various secreted chemotactic factors [21]. 

Therefore, research into other mechanisms in which MSCs could exert their effect began, and it is now believed that it is the paracrine “trophic activity” [22] of MSCs that provides the regenerative microenvironment. The secretion of bioactive materials by MSCs in response to injury mitigates the inflammatory response and in turn decreases injury and promotes repair [22, 23]. This secretion acts not only by directly initiating intracellular pathways but also by indirectly inciting another cell in the area to secrete a functionally active agent [24]. Inhibition of apoptosis, scar formation and promotion of angiogenesis, as well as stimulation of injured tissue to differentiate into regenerative units, are the downstream results of such bioactive agents.

### 1.2. Immunomodulatory Response

More recently, MSCs have also been shown to have an *in vivo* immunomodulatory effect, most notably in graft versus host disease [11, 25]. Extensive *in vitro *studies demonstrate that MSCs lead to inhibition of TNF-*α* and INF-*α* production with an increase in IL-10, thereby limiting Tcell expansion [22]. Via prostaglandin E_2_, MSCs can inhibit natural killer cell proliferation and cytotoxicity *in vitro*, as well as steer monocytes and mature dendritic cells to an immature dendritic cell state, rendering them more susceptible degradation by natural killer cells [25].

Another group looked at the response of MSCs to the proinflammatory cytokine IFN-*γ* or in combination with TNF, IL-1*α*, and IL-1*β* and found that MSCs secreted chemoattractants for T cells as well as inducible nitric oxide synthetase. The subsequent production of nitric oxide inhibited the T cell activation [26]. However, this same mechanism of T cell suppression was not found across all species, and while human and monkey MSCs did not induce nitric oxide synthetase, T cells were still suppressed in a different manner [26].

It is likely a combination of the paracrine activity of the MSCs and direct cell contact that allows MSCs to have both a reparative and immunomodulatory effect. Their response to injury in the absence of invading organisms produces a negative feedback loop to hinder the otherwise excessive inflammatory and immune response produced in many disease states [18]. It is from this knowledge that many of the clinical experiments both *in vitro* and *in vivo* have evolved and contributed to the breadth and variety in which MSCs are being applied.

## 2. Mesenchymal Stem Cells in the Bladder

As research into utilizing MSCs has moved forward, extensive information has been obtained into the venues in which MSCs can be applied. The application of MSCs has been well established in several organ systems, most notably musculoskeletal, vascular, and reticuloendothelial, however it seems that less has been done in the genitourinary system. In this paper, we will focus on the research and applications of MSCs specifically in the bladder. 

Tissue engineering was first initiated in bone remodeling, with the concept of applying MSCs to a scaffold and implanting into bony repair sites [22]. In the bladder, initial interest has focused on tissue regeneration in hopes of allowing an autologous bladder augmentation and circumventing the multiple morbidities associated with enterocystoplasty. However, as more information regarding differentiation potential, successful gene therapy, and immunomodulation has progressed, the number of potential applications of MSCs to bladder research has increased as well.

### 2.1. Tissue Regeneration

The ideal application of bladder tissue regeneration is to develop a functional urinary bladder for patients with either congenital or acquired bladder defects. Currently, this means augmenting a poorly compliant, fibrotic bladder, reconstructing a partially removed bladder or actually creating a reservoir from intestine. Without surprise, MSCs have found their way into this arena and currently are showing extreme promise as a source of cells for graft development.

An important advance in tissue regeneration was determining that MSCs could be induced into tissue specific differentiation. MSCs cultured in conditioned mediums acquire a smooth muscle cell phenotype, staining positively for alpha-smooth muscle actin, myosin, and calponin [27, 28]. Culturing in the presence of other myogenic growth factors can also lead to a phenotypic profile of smooth muscle [27, 29]. Urothelium can also induce mesenchyme into smooth muscle differentiation, [30] a property that will be extremely useful for future in vivo MSC studies. 

Previously it was thought that MSCs only differentiated into stromal tissues. However, recent reports are encouraging that in appropriate environments, MSCs are capable of endodermal differentiation as well [9, 31, 32]. Xenografts of MSCs and embryonic bladder mesenchyme were incubated under a renal capsule and after 6 weeks showed a bladder structure, including both MSC-derived urothelium and smooth muscle with a lumenoid cavity [31]. Tian et al. were also able to induce urothelial differentiation from MSCs when grown in urothelial cell-conditioned medium [9]. Another group was surprised to find marked urothelial cells on an MSC seeded SIS (small intestinal submucosa) graft in a porcine bladder augmentation suggesting that MSCs transdifferentiated into the urothelial cells [33].

Tissue regenerative studies involving the bladder have primarily focused on using an underlying matrix that is either seeded with cells or left unseeded for in vivo infiltration [9, 27, 34, 35]. Initial attempts have been hindered by poor scaffold materials and cells that do not contribute to creating the smooth muscle component necessary for contractility and compliance that characterizes the bladder. MSCs, for many reasons previously discussed, are emerging as an extremely promising cell population for tissue regeneration. Kanematsu et al. transplanted GFP-labeled MSCs into lethally irradiated rats and determined the recruitment of MSCs to an acellular matrix graft on the dome of the rat bladder eight weeks later. Within two weeks the MSCs were seen to be populating the graft and twelve weeks later had not only reconstituted the smooth muscle cell layer but also induced native smooth muscles cells to infiltrate the graft as well [27]. Recent investigations have looked at biodegradable scaffolds including small intestinal submucosa [36] (SIS), elastomeric poly(1,8 octanediol-co-citrate) based thin films [35], and 3D nanofibrous scaffolds [34] seeded with bone marrow-derived stromal cells with favorable results. 

After identifying SIS as a suitable matrix for cell seeding and bladder augmentation [37], Zhang et al. compared MSC-seeded SIS to bladder smooth muscle cell-seeded SIS both in vitro and in vivo and showed that MSCs had similar cell proliferation, contractile phenotype and histological appearance to smooth muscle cells [36]. The grafts showed excellent penetration of the MSCs with positive staining for alpha-smooth actin. However, only two of the augments did not show shrinkage (one MSC seeded, one smooth muscle cell seeded) limiting the in vivo functional interpretation of this study. Bladder reconstitution was improved and noted earlier in SIS bladder augments in a rat model [38].

Tian et al. were able to differentiate MSCs into smooth muscle cells on a nanofibrous 3D poly-L-lactic acid scaffold. The porous structure allows a favorable microenvironment for SMC differentiation and regeneration and even showed capillary formation after one month of subcutaneous incubation [9, 34]. Since MSCs have identified their potential as a cell source for seeding scaffolds, the search continues for the scaffold that is the most functionally equivalent to the bladder. Sharma et al. recently evaluated a novel synthetic elastomeric scaffold that was seeded with MSCs and augmented onto rat bladders [35]. After 10 weeks the harvested graft showed a robust, trilayered architecture with retained pliability compared to the unseeded graft [35]. The urothelial layer consisted of local ingrowth from the native rat bladder; however, well-formed smooth muscle bundles were regenerated from MSCs. Furthermore, it is felt that MSC-pseeded scaffolds that allow for repeated contraction and expansion contribute to the maturation of the MSCs by stimulating smooth muscle differentiation [34, 35]. All of these results are promising, and there is still exceptional enthusiasm in the realm of bladder tissue engineering with MSCs as donor cells at the forefront.

### 2.2. Repair and Fibrosis

As exciting as it seems to use MSCs for de novo tissue regeneration, more promising is the application of MSCs in tissue remodeling and repair. Several models of fibrosis in lung, heart, and liver have utilized MSCs in modulating the fibrotic response. Rats treated with carbon tetrachloride (CCL_4_) or dimethylnitrosamine (DMN) to induce an experimental liver fibrosis similar to humans were systemically administered MSCs. Compared to controls, rats treated with MSCs showed not only and improvement in survival but also an improvement in collagen deposition, fibrotic index, an improved liver function [15]. Ortiz et al. evaluated the fibrotic response of lung tissue to bleomycin with and without treatment of MSCs [19]. Again noted was an improvement in inflammation and fibrosis in response to bleomycin in MSC-treated mice. Mice treated with MSCs were also noted to decrease expression of matrix metalloproteinases 2 and 9. In both models, earlier administration of MSCs after the insult leads to the greatest improvement in fibrosis.

Very little research has been performed investigating the recruitment of MSCs to an injured bladder model. Bladder fibrosis is an untoward effect of bladder outlet obstruction which can affect both children and adults. In a partially obstructed bladder outlet model, Tanaka et al. showed recruitment of bone marrow-derived cells in both the urothelium and stromal layers [39]. GFP-labeled fetal liver cells were used to repopulate irradiated bone marrow in mice, and GFP-positive bone marrow-derived cells were isolated in fibrotic bladders after 12 weeks of partial obstruction. There was an increase in expression of chemokine CCL2 in the obstructed bladders which has been associated with fibrosis in other organs. This research sets up a platform for further investigation into the role of MSCs in fibrotic conditions of the bladder and for determining key factors in the recruitment of MSCs to the injured bladder. 

A surprise outcome of improved remodeling after cryo-induced injury to the bladder was noted in a study that was looking for amniotic fluid MSCs and bone marrow MSCs differentiation into smooth muscle cells [40]. The group directly transplanted the MSCs into the site of injury in the rat bladder, but only found a very small amount of MSCs differentiated into the smooth muscle cells that contributed to the repair process. Instead they found a reduction in the hypertrophy that generally results from the regeneration after such an injury [40]. Their results further support the shift in role of MSCs in repair from engraftment and differentiation to a support and mediator.

### 2.3. Gene Therapy

Another application of MSCs has been in the realm of genetic therapy. MSCs have emerged as potential vehicles for the delivery of transgenes because of their characteristic tissue specific homing, long life span, and low potential for immunologic response [13]. Among other methods, viral vectors are used in the introduction of the transgenes. Lentivirus-transduced murine MSCs were able to maintain their *in vitro* multipotency and the, MSCs were subsequently identified *in vivo *in minimally injured transplanted mice [41]. Gene-modified MSCs are being utilized *in vivo* in a variety of inherited neurologic and blood disorders as well as in cardiovascular disease, musculoskeletal disease, and tumor growth [13, 42, 43].

A study using MSCs in an acute lung injury model also showed that MSCs injected after endotoxin injury showed a reduction in edema, vascular injury, and pulmonary hypertension [44]. Another group proceeded to infect MSCs with angiopoietin-1 (Ang-1), an important mediator in the pathologic changes that occur in the lung in response to injury [45]. Mice that were treated with MSC-Ang-1 showed significant improvement even over mice treated with MSCs alone in regards to lipopolysaccharide-induced lung injury. Increased expression of Ang-1 was noted in mice treated with the transfected MSCs and persisted for 14 days until sacrifice [45].

Transcribing these models to bladder fibrosis, antitumor therapy or interstitial cystitis is certainly not difficult to envision. Increased expression of a substance further capable of modulating the extracellular matrix may improve the already postulated attenuation of fibrosis created by MSCs. Potential for targeted chemotherapy to prevent recurrences of superficial bladder cancer could replace intravesical therapy. Gene therapy for improving the glycosaminoglycan (GAG) layer in urothelium in patients with interstitial cystitis could provide further information to the etiology of a confounding clinical problem.

## 3. Conclusion

MSCs continue to allure researchers with up to 5–10 papers a day regarding MSCs being published. Further elucidation of the biochemical pathways that lead to their specific homing to various injured sites will be crucial in the trajectory of future research efforts. Standardization in the culturing techniques and identification of injected MSCs will be necessary for replication of work. In the specific realm of the genitourinary system, MSCs hold much promise for the future of tissue engineering, treatment of fibrotic bladders, and gene therapy.
